# The Effects of Extracorporeal Shock Wave Therapy on Spastic Muscle of the Wrist Joint in Stroke Survivors: Evidence From Neuromechanical Analysis

**DOI:** 10.3389/fnins.2020.580762

**Published:** 2021-01-21

**Authors:** Yan Leng, Wai Leung Ambrose Lo, Chengpeng Hu, Ruihao Bian, Zhiqin Xu, Xiyao Shan, Dongfeng Huang, Le Li

**Affiliations:** ^1^Department of Rehabilitation Medicine, The First Affiliated Hospital, Sun Yat-sen University, Guangzhou, China; ^2^Department of Rehabilitation Medicine, The Seventh Affiliated Hospital, Sun Yat-sen University, Shenzhen, China; ^3^Institute of Medical Research, Northwestern Polytechnical University, Xi'an, China

**Keywords:** stroke, spasticity, extracorporeal shock ware therapy, muscle mechanical properties, upper extremities, neurorehabilitation

## Abstract

**Background:** This study combined neuromechanical modeling analysis, muscle tone measurement from mechanical indentation and electrical impedance myography to assess the neural and peripheral contribution to spasticity post stroke at wrist joint. It also investigated the training effects and explored the underlying mechanism of radial extracorporeal shock wave (rESW) on spasticity.

**Methods:** People with first occurrence of stroke were randomly allocated to rESW intervention or control group. The intervention group received one session of rESW therapy, followed by routine therapy which was the same frequency and intensity as the control group. Outcome measures were: (1) NeuroFlexor method measured neural component (NC), elastic component (EC) and viscosity component (VC), and (2) myotonometer measured muscle tone (F) and stiffness (S), (3) electrical impedance myography measured resistance (R), reactance (X) and phase angle (θ); (4) modified Asworth scale; (5) Fugl Meyer Upper limb scale. All outcome measures were recorded at baseline, immediately post rESW and at 1-week follow-up. The differences between the paretic and non-paretic side were assessed by *t*-test. The effectiveness of rESW treatment were analyzed by repeated-measures one-way analysis of variance (ANOVA) at different time points.

**Results:** Twenty-seven participants completed the study. NC, EC, and VC of the Neuroflexor method, F and S from myotonometer were all significantly higher on the paretic side than those from the non-paretic side. R, X, and θ from electrical impedance were significantly lower on the paretic side than the non-paretic side. Immediately after rESW intervention, VC, F, and S were significantly reduced, and X was significantly increased. The clinical scores showed improvements immediate post rESW and at 1-week follow-up.

**Conclusions:** The observed changes in upper limb muscle properties adds further support to the theory that both the neural and peripheral components play a role in muscle spasticity. ESW intervention may be more effective in addressing the peripheral component of spasticity in terms of muscle mechanical properties changes. The clinical management of post stroke spasticity should take into consideration of both the neural and non-neural factors in order to identify optimal intervention regime.

## Introduction

Spasticity is a clinical sign frequently appears in patients with stroke (Katoozian et al., 2018). Spasticity contributes to complications such as pain, altered posture, anchylosis or deformities and osteoporosis which affects the recovery of motor function and quality of lives of stroke survivors (Malhotra et al., 2011). The stretch reflex arc impairment has been considered as a main contributing factor to spasticity. However, the structural and component changes in muscle fibers and tendon, including muscle atrophy (e.g., fiber size reduction, fiber loss, lean muscle decline), muscle cross-sectional area shrinking and intramuscular fat accumulation, along with concomitant mechanical or morphological alterations of the intra- and extracellular components also played an important role in the development of spasticity (Lieber et al., 2004; Li and Francisco, 2015). The current management regimens for spasticity include physical therapy (such as neuromuscular stimulation, functional electrical stimulation, or ultrasound therapy), oral anti-spasticity drugs, intrathecal baclofen, chemical nerve block and motor point block. However, spasticity cannot always be adequately managed despite the diversity of treatment regimens. The common side effects of drugs and the invasiveness of local treatment are undesirable. Thus, effective and non-invasive intervention methods for spasticity are urgently needed, particularly for the intervention that target the peripheral muscular factor that contributes to muscle spasticity (Yelnik et al., 2010). The application of quantitative evaluation techniques of muscle properties will facilitate the understanding of the underpinning mechanisms and the effectiveness of new intervention strategy of post stroke spasticity management (Lindberg et al., 2011; Chuang et al., 2012; Pennati et al., 2016).

Previous studies showed that extracorporeal shock wave (ESW) could increase the efficiency of the tissue regeneration process by promoting blood microcirculation and tissue rheology (Goertz et al., 2012; Link et al., 2013). Other study demonstrated the neuronal effects of ESW therapy to increase the expression of neuronal nitric oxide synthase and creates new axons (Lee and Kim, 2015) which indicated that ESW might be a promising modality to treat spasticity caused by the upper motor neuron (UMN) lesion non-invasively(Manganotti and Amelio, 2005; Sohn et al., 2011). Based on the propagation pattern, there are the two different types of ESW stimulus: focused extracorporeal shock wave (fESW) and radial extracorporeal shock wave (rESW). The fESW is generated electromagnetically, electrohydraulically and piezoelectrically with rapidly increased pressure that means more invasive with the highest energy exposure in the focal area of deep zones. The pressure of rESW is pneumatically generated by the ballistic device that slowly increases. The wave is absorbed to a depth of 3 cm which is less invasive and better tolerance (Dymarek et al., 2014, 2016a). Previous studies reported that both types of ESW could successfully treat spasticity. Significant effectiveness of ESW was observed in patients suffering from cerebral palsy (Amelio and Manganotti, 2010), multiple sclerosis(Marinelli et al., 2015) and stroke (Daliri et al., 2015). ESW therapy was considered as a useful tool to treat spasticity and to improve joint range of motion and gait pattern in neurorehabilitation. It was reported that ESW could reduce pain and muscle tone in MS patients through acting on the non-reflex hypertonia, such as reducing muscle fibrosis. A total of six studies (Manganotti and Amelio, 2005; Santamato et al., 2013; Troncati et al., 2013; Daliri et al., 2015; Li et al., 2016; Wu et al., 2018) that utilized ESW intervention to treat upper limb spasticity in stroke patients reported significant reductions in MAS, increased of elbow passive range of movement and Fugl-Meyer score (Wu et al., 2018). The authors of these studies concluded that ESW therapy was at least as effective as botulinum toxin type A (BoNT-A) for the treatment of post stroke upper limb spasticity. Daliri et al. (2015) reported a significant improvement in post-stroke spasticity of the wrist flexor muscles as assessed by the α motor neuron excitability and the H-reflex (HMR, Hmax/Mmax ratio) of electromyography (EMG) post ESW intervention. However, Manganotti et al. (Manganotti and Amelio, 2005) reported no significant alterations on the motor nerve conduction parameters of nEMG examination after ESW treatment. In two studies that investigated the effect of ESW treatment on lower limb post-stroke muscles spasticity, the authors reported no statistically significant change in all EMG parameters (F wave, H-reflex, and H/M ratio) was observed. These findings confirm that the effect of ESW is not related to spinal excitability modifications (Sohn et al., 2011; Santamato et al., 2014). These contradictory results cast some uncertainties on the underpinning mechanism of ESW intervention for spasticity, which add some support to the hypothesis that ESW affects rheological properties of the hypertonic muscles, in particular on its impact on altering the periphery biomechanical properties of muscles that contribute to spasticity. This is partly related to the current status of the application of clinical scales for subjective measurements, or rely on a single type of assessment method to evaluate spasticity.

A recently published review discussed several advanced quantitative measurement technologies to assess spasticity (Luo et al., 2019). The authors emphasized the importance to apply different measurement techniques to assess spasticity quantitatively and objectively, given the neural and peripheral components that contribute to spasticity. It is therefore logical to assess the mechanism of ESW intervention with a combination of assessment techniques to clarify its effects on the neural and peripheral contribution of muscle spasticity. The Neuroflexor (NF) method is a recently developed instrument based on the biomechanical modeling method (Lindberg et al., 2011; Gäverth et al., 2013). Resistant force during passive extension of the wrist joint was measured to estimate the neural and non-neural contribution to spasticity. Three parameters are derived: neural component (NC), elasticity component (EC), and viscosity component (VC) (Lindberg et al., 2011; Gäverth et al., 2013). The NF was validated in people with Parkinson's disease, cerebral palsy and stroke (Gäverth et al., 2014; Zetterberg et al., 2015; Kachmar et al., 2016). Our previously published study confirmed the feasibility to assess muscle stiffness, elasticity and viscosity characteristic of upper extremity spastic muscle in patients with stroke using the NF technique (Leng et al., 2019). A systematic review published recently encouraged the application of measuring technique to, including myotonometer, to assess the changes of muscle properties induced by ESW in spastic muscle (Dymarek et al., 2020). This would facilitate further understanding and new insights into the mechanism that underpins the intervention.

Myotonometer is a handheld instrument to objectively quantify muscle biomechanical properties. It applies multiple short impulses over the muscle bulk via the testing probe to generate vertical oscillations in the muscle fibers (Gapeyeva and Vain, 2008). The oscillation waveform is reflective of the viscoelastic properties of the muscle including muscle tone, elasticity, and stiffness (Gapeyeva and Vain, 2008; Lo et al., 2017). Published studies have indicated that the myotonometer is reliable and valid to measure skeletal muscle viscoelastic parameters in individuals with stroke (Rydahl and Brouwer, 2004; Chuang et al., 2012; Lo et al., 2017). Electrical impedance myography (EIM) is considered as a biomarker to assess neuromuscular disease progress and response to therapy. EIM measures the inherent muscle properties by sending an alternating sine wave current to the tissue and detecting the surface voltage, acquiring the parameters of resistance (R), reactance (X), and Phase angle [θ = arctan(X/R)]. These parameters are associated with the muscle's component, extracellular and intracellular fluids, the cell membrane integrity, and tissue interfaces, respectively (Esper et al., 2006). EIM is supposed to reflect on muscle composition and structure rather than its electrical activity (Tarulli et al., 2006). To date, there is limited number of study that investigated the changes of mechanical and electrical properties after ESW intervention in stroke survivors (Lo and Li, 2020).

Therefore, the present study aimed to assess the neural and peripheral contribution to spasticity post stroke by combining biomechanical modeling method with mechanical muscle properties and muscle composition information from electrical impedance measurement. This was followed by an investigation of the effects and underlying mechanism of rESW on spasticity.

## Materials and Methods

### Study Design

**Figure 1:**
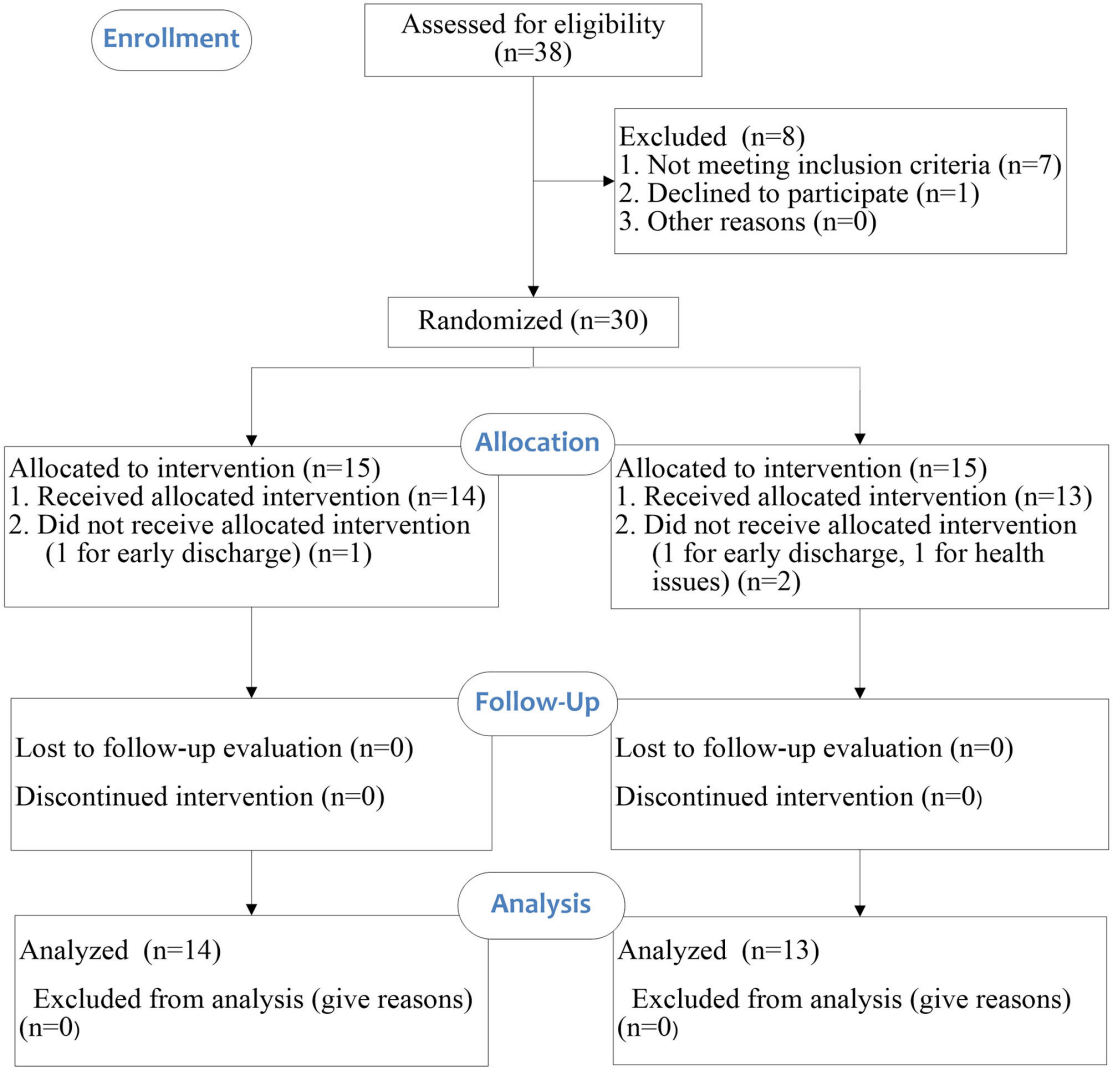
CONSORT flow diagram.

This study was a single-blinded randomized controlled study. All participants were randomly allocated to ESW intervention group or control group. An overview of the study is illustrated in the CONSORT flow diagram (Figure 1). Data collection took place at the Department of Rehabilitation of the First Affiliated Hospital of Sun Yat-Sen University between June 2019 and January 2020. A randomization schedule was pre-generated in SPSS by an independent statistician. The sequence of allocation was kept in sealed envelope. The randomization process allocated each participant an identification number which appeared on all report forms to maintain confidentiality.

### Sample Population

The inclusion criteria were as follows: (1) the first occurrence of stroke as confirmed by computed tomography or magnetic resonance imaging which resulted in unilateral hemiparesis; (2) at least 1 month of stroke onset; (3) the passive range of wrist joint was between −20° palmar flexion and 30° dorsiflexion; and (4) the MAS score for the radiocarpal joint ≥1. The exclusion criteria were as follows: (1) medically unstable; (2) receiving muscle relaxant or anti-spastic medication; (3)infraction at the cerebellum region; (4) coagulation disorders, electronic and metal implants or skin lesion of the upper extremity; (5) cognitive dysfunction as assessed by Mini Mental State Examination (score >26); (6) upper limb fracture or non-muscle spasm related restriction of joint movement.

### Ethics Consideration

This study was approved by the Ethics Committee of the First Affiliated Hospital, Sun Yat-sen University [Ethics Number: (2017).143]. In addition, the trial had been prospectively registered at Chinese Clinical Trial Registry (ChiCTR- IOR-17012299). All participants provided written consent prior to enrollment. Study procedures were conducted according to the Declaration of Helsinki.

### Outcome Measures

Outcome measures were recorded by independent assessors who were blinded to the group allocation of participants. Neural and peripheral contribution of spasticity were assessed by the parameters of Neural component (NC), Elastic component (EC) and Viscosity component (VC) measured by the NeuroFlexor. Mechanical muscle properties of tone (F) and stiffness (S) were assessed by myotonometer. EIM measured the parameters of resistance (R), reactance (X), and Phase angle (θ) [θ= arctan (X/R)]. Severity of muscle spasm was clinically assessed by the Modified Ashworth Scale (MAS). Upper limb function was assessed the Fugl-Meyer Assessment (FMA) scale.

### Study Procedures

Anthropometric characteristics of the sample population including age, gender, paretic side and stroke onset time, were collected before the experiment began. Baseline assessment was then conducted (t0). For the intervention group, ESW was administered immediately after baseline assessment and outcome measures were recorded right after ESW (t1). Participants then received 1 week of routine rehabilitation therapy at the same frequency and intensity with the control group. Participants in the control group received 5 sessions of regular rehabilitation treatment within a week. Each session lasted for 1.5 h and included stretching exercise therapy, occupational therapy and neurodevelopmental facilitation techniques. Outcome measures were then recorded immediately by the end of the intervention period (7 days) for both groups (t2).

### Instruments

#### NeuroFlexor

**Figure 2:**
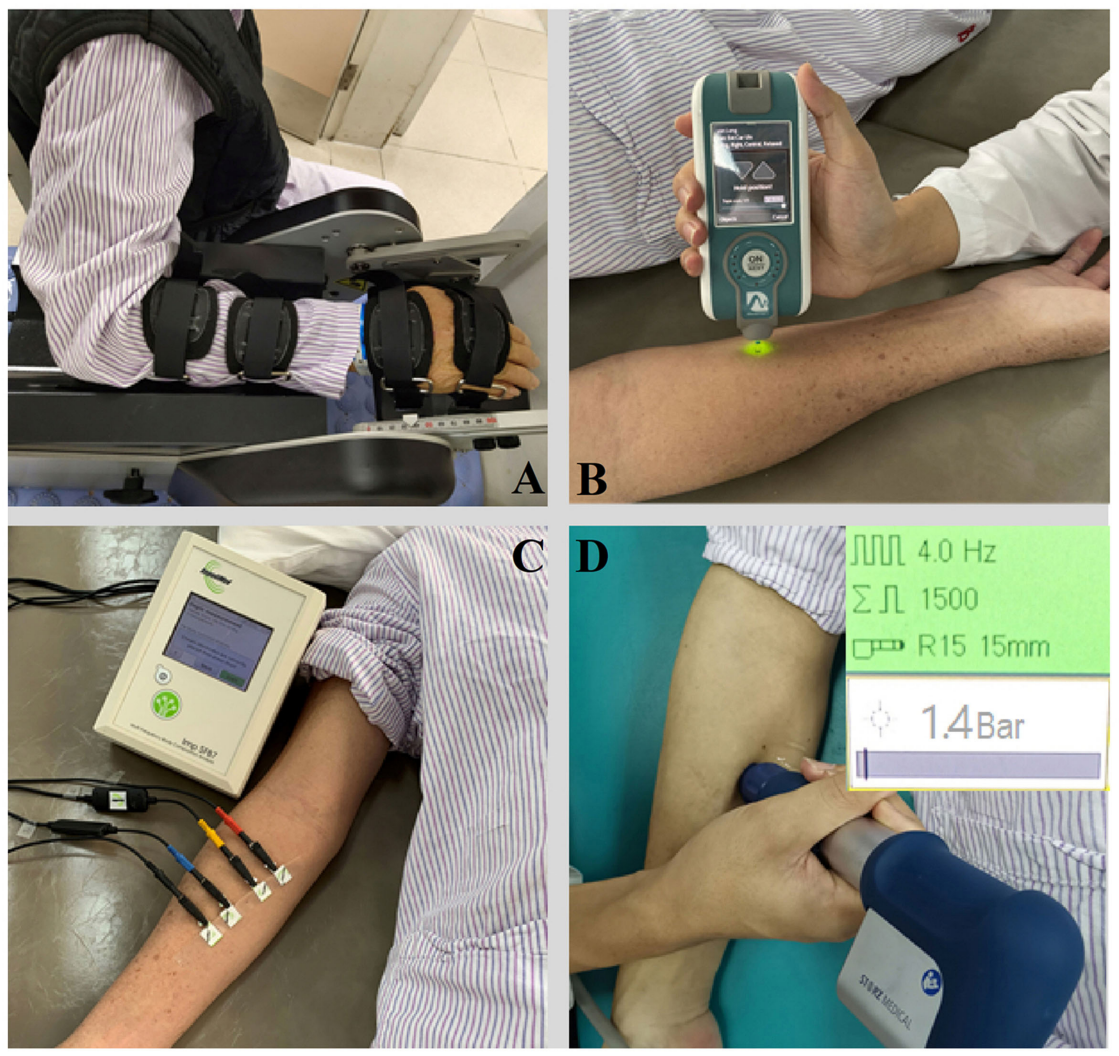
The apparatus for measurement and therapy adopted in the study. **(A)** NeuroFlexor; **(B)** Myotonometer; **(C)** Electrical impedance myography; **(D)** Extracorporeal shock wave therapy.

The NeuroFlexor (Aggro MedTech AB, Solna, Sweden) produces passive movement at constant speed and records the passive resisting forces from the wrist joint in real time by the force sensor under the moveable platform (Figure 2A). During passive joint movement, time, angle and resisting forces were recorded simultaneously. The NF instrument ran 7 times at slow mode of 5°/s and 12 times at fast mode of 236°/s. The range of wrist movement was 50° with a starting position at −20° palmar flexion and an end position at 30° extension (7, 21). Three components of NC, EC and VC were calculated based on a biomechanical model which was described in detailed in previous study (Lindberg et al., 2011; Pennati et al., 2016; Leng et al., 2019). EC represents the length-dependent resistant force and VC represents the viscosity-dependent resistant force. NC is estimated at maximal extension at the end of the fast passive movement by subtracting the EC and VC from the total force.

#### Myotonometer

A handheld myotonometer (MyotonPRO®, Estonia) was used to quantify the flexor carpi radialis muscle tone and stiffness. The test location as the thickest part of the flexor carpi radialis, which originates from the medial epicondyle of the humerus and inserts at the surface of the palm at the base of the second metacarpal bone (Garten, 2013). The patient laid in supine with upper extremity relaxed on the side. The wrist joint was kept in the neutral position with finger slightly flexed. B-mode ultrasound scan was then conducted to confirm the location of the thickest part of the muscle bulk. The testing probe of the myotonometer was placed perpendicularly to the skin surface of the tested location. The probe was first loaded by pushing against the skin surface to the required depth. Once the required depth was reached (indicated by a change of indicator light from red to green), the device then applied three short impulses (1 s apart) to induce damped oscillations within the muscle bulk (Figure 2B). The oscillation pattern recorded by the transducer was used to calculate the muscle mechanical properties of tone and stiffness (Lo et al., 2017, 2019).

#### Electrical Impedance Myography

The muscle electrical resistance properties that reflect the muscle composition and structure of the flexor carpi radialis was assessed by EIM (Imp SFB7 Impedimed, Inc., Sydney, NSW, Australia). The center point of the electrodes was identified as the upper third between the medial epicondyle of the humerus and the second metacarpal bone at the radial side of the wrist. B-mode ultrasound scan was conducted to confirm the location of the muscle belly and the direction of the muscle fibers of flexor carpi radialis. Two pairs of electrodes were linearly arranged along the muscle fibers direction, including one pair of voltage electrodes on the inner regions and an outer pair of current electrodes (30). Each pair of electrodes was distributed symmetrically along the center point marked in advance. The distance between the two outer current electrodes and the two inner voltage electrodes was 60 and 20 mm, respectively. The dimension of the electrodes was 13 × 10 mm (Figure 2C). Three measurements were recorded at each assessment and the mean value of the measurement was used for statistical analysis. The data obtained by the device was exported for offline analysis by the bespoke software Bioimp. The parameters of resistance, reactance, and phase angle were recorded across multiple frequencies of between 5 and 1,000 kHz, and those parameters obtained at 50 kHz was chosen to analysis.

#### Extracorporeal Shock Wave

A radial ESW (rESW) pneumatic device (Chattanooga, DJO Global Inc., Guildford, United Kingdom) was adopted to provide a single session of shock wave intervention to the radial carpi flexor muscle (Figure 2D). The treatment protocol of ESW was follow: 1,500 shots with a pressure of 1.5 bars and wave irradiation of 4 Hz (Dymarek et al., 2020). The treating area was focused on the muscle belly of the radial carpi flexor. The energy applied was 0.038 mJ/mm^2^.

### Statistical Analysis

Statistical analyses were conducted using SPSS 22 (IBM, United States). Descriptive statistics were calculated for all dependent variables. The data between the experimental group and the control group were analyzed with independent *t*-test or corresponding non-parametric test. The differences of all recorded parameter between the paretic and non-paretic side were assessed by independent *t*-test. The correlations between the different parameters as measured by difference device and clinical outcome measures were determined using Pearson correlations for normally distributed data, or Spearman's correlations for non-normally distributed data. The effectiveness of rESW treatment in the intervention group at different time points were analyzed by repeated-measures one-way analysis of variance (ANOVA) and LSD *post-hoc* test. The significant level of all statistical tests was set at 0.05.

## Results

Thirty stroke survivors participated in the current study and 27 of them completed all assessments. Three participants (one from the intervention group and two from the control group) did not complete the final assessment (t2) due to personal reasons. The clinical characteristics and functional levels of the sample population are presented in Table 1. There was no significant difference between the intervention group and control group at baseline.

**Table 1:** The characteristic of the sample populations.

**Characteristic**	**Experimental group**	**Control group**	**Inter-group *P*-value**
Patients (*n*)	14	13	
Age (years)	51.14 ± 13.68	58.92 ± 10.08	0.107
Gender (M/F)	11/3	11/2	0.686
Weight (kg)	64.21 ± 10.53	66.08 ± 7.01	0.596
Height (m)	1.65 ± 6.90	1.69 ± 5.03	0.141
Stroke subtype (IS/HS)	8/6	10/3	0.276
Affected site(R/L)	9/5	10/3	0.472
Time since onset (months)	17.39 ± 29.18	24.42 ± 37.09	0.588
MAS (score)	2.00 ± 0.78	1.85 ± 0.80	0.268
FMA (score)	22.79 ± 14.37	30.23 ± 20.73	0.285

F, female; M, male; R, right; L, left; IS, ischaemicstroke; HS, haemorrhagicstroke; n, number. FMA, Fugl-Meyer assessment scale of the motor function in paretic upper-extremity; MAS, Modified Ashworth Score. ^*^Statistically significant between experimental group and control group.

### Bilateral Differences

**Figure 3:**
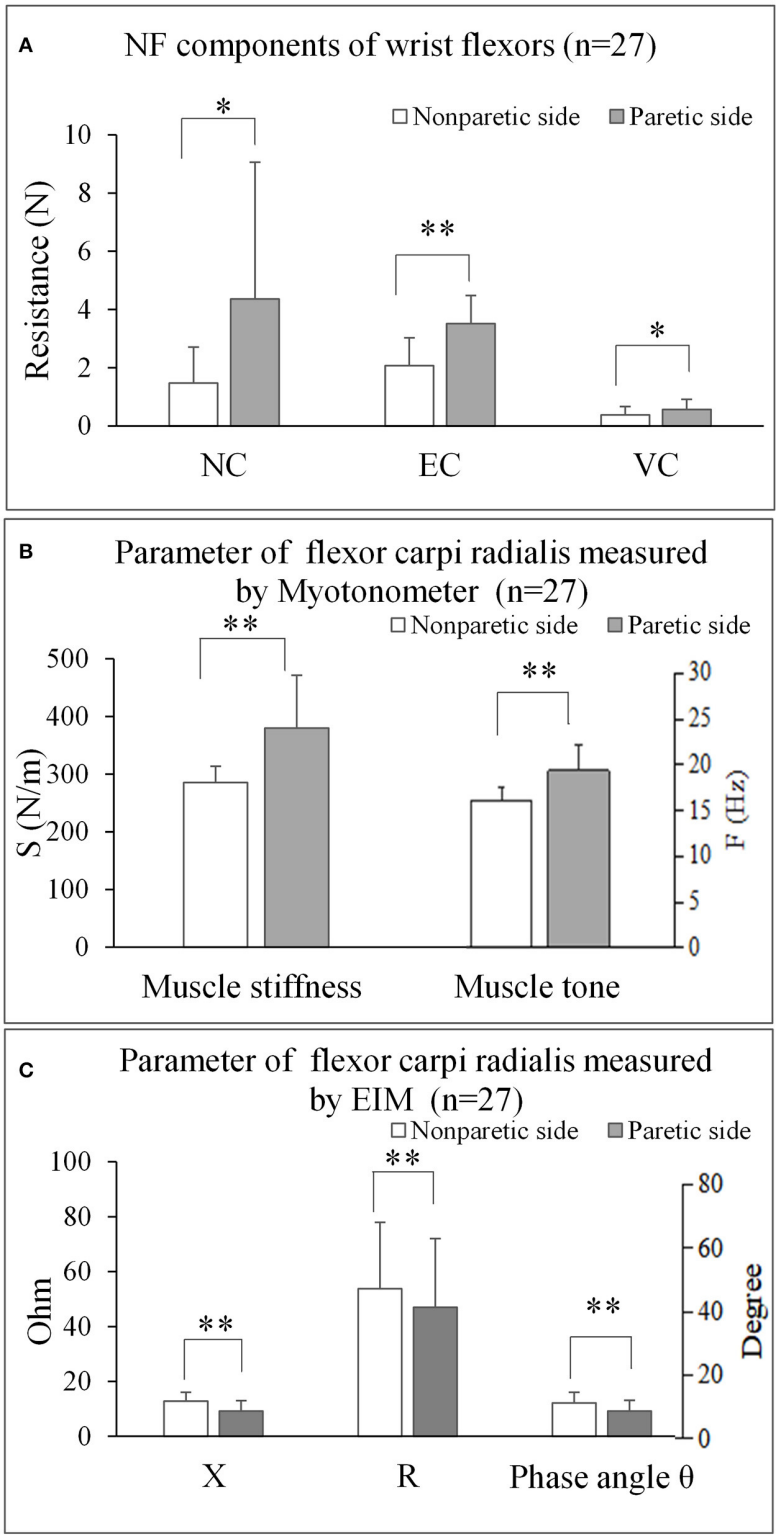
The comparisons of bilateral parameters for each measuring techniques. **(A)** NF components of wrist flexors (*n* = 27). **(B)** Parameter of flexor carpi radialis measured by Myotonometer (*n* = 27). **(C)** Parameter of flexor carpi radialis measured by EIM (*n* = 27). NF, NeuroFlexor; NC, Neural Component; EC, Elasticity Component; VC, Viscosity Component; S, Stiffness; F, Frequency; EIM, Electrical impedance myography; X, Reactance; R, Resistance. *P < 0.05; ** *P* < 0.001.

The three parameters of NC, EC, and VC, on the paretic side were significantly higher than the non-paretic side (*p* < 0.05) (Figure 3A). The myotonometer measured muscle tone and stiffness on the paretic side were significantly higher than the non-paretic side (*p* < 0.001) (Figure 3B). The EIM measured parameters of resistance, reactance and phase angle were significantly lower on the paretic side than the non-paretic side (*p* < 0.001) (Figure 3C). Table 2 shows a summary of the NF, myotonometer and EIM parameters on the paretic and non-paretic sides.

**Table 2:** Summary of the NF, Myotonometer and EIM parameters on the paretic and non-paretic side.

**Measurements**	**Paretic side**	**Non-paretic side**	**Inter-group *P*-value**
**NeuroFlexor**			
NC (N)	4.26± 4.70	1.47 ± 1.24	0.007^*^
EC (N)	3.51 ± 0.97	2.07 ± 0.96	<0.001^**^
VC (N)	0.57± 0.34	0.38 ± 0.29	0.041^*^
**Myotonometer**			
S (N/m)	379.96 ±91.32	285.19 ± 28.37	<0.001^**^
F (Hz)	19.34 ± 2.93	16.03 ±1.61	<0.001^**^
**EIM**			
R (Ohm)	47.05 ±25.04	53.78 ± 24.21	<0.001^**^
X (Ohm)	9.18± 3.82	12.85 ± 3.27	<0.001^**^
Phase angle (Degree)	9.25 ± 3.85	12.23 ±3.91	<0.001^**^

*NC, Neural Component; EC, Elasticity Component; VC, Viscosity Component; S, Stiffness; F, Frequency; EIM, Electrical impedance myography; X, Reactance; R, Resistance*.

* P < 0.05;

** *P < 0.001*.

### Correlation Between Parameters

**Figure 4:**
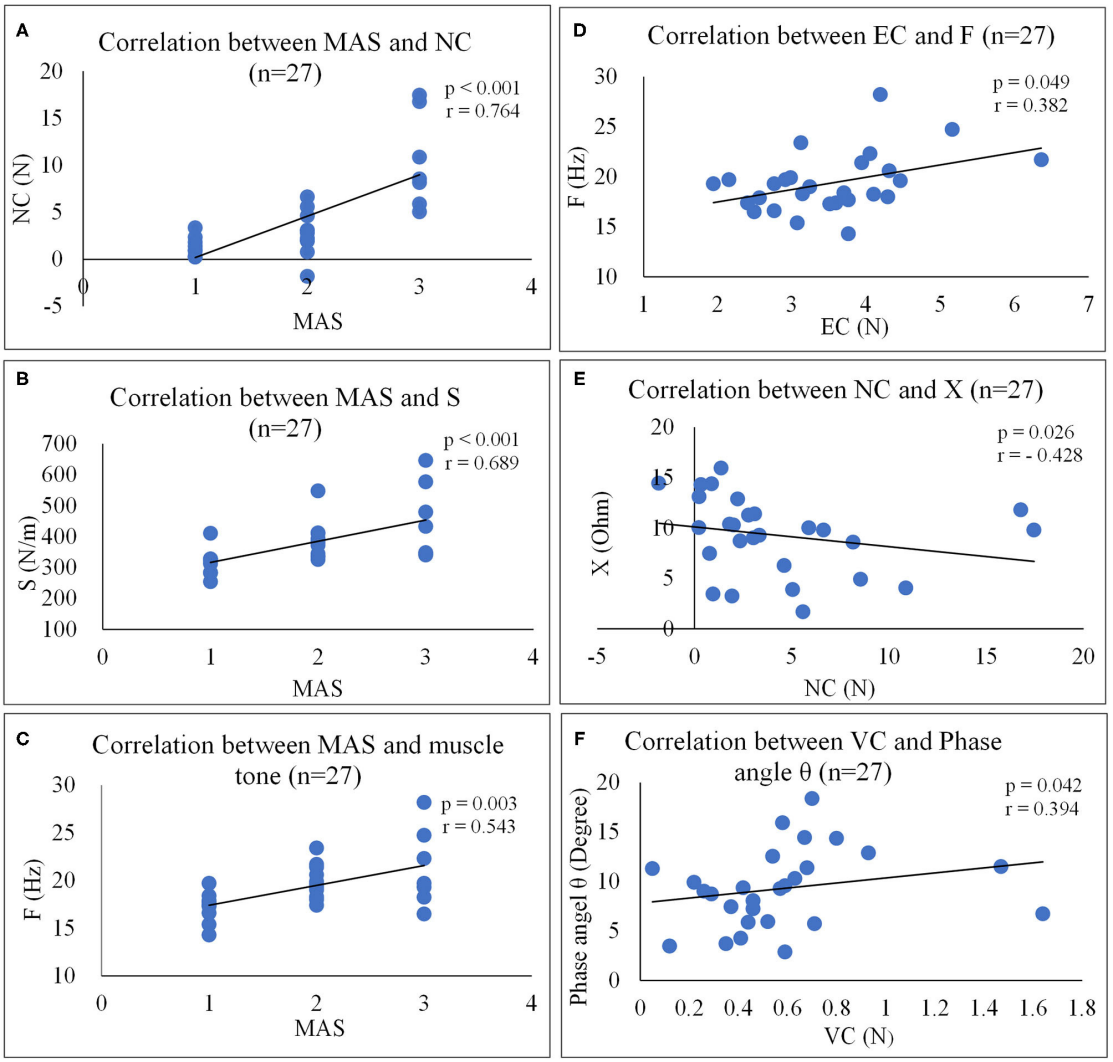
Correlation analyses between outcome measures. **(A)** Correlation between MAS and NC (*n* = 27). **(B)** Correlation between MAS and S (*n* = 27). **(C)** Correlation between MAS and muscle tone (*n* = 27). **(D)** Correlation between EC and F (*n* = 27). **(E)** Correlation between NC and X (*n* = 27). **(F)** Correlation between VC and phase angle θ (*n* = 27). NF, NeuroFlexor; NC, Neural Component; EC, Elasticity Component; VC, Viscosity Component; S, Stiffness; F, Frequency; EIM, Electrical impedance myography; X, Reactance; R, Resistance.

Moderate to high positive correlations were observed between the MAS and NC (Figure 4A, *r* = 0.764, *p* < 0.001), between MAS and S (Figure 4B, *r* = 0.689, *p* < 0.001) and between MAS and F (Figure 4C, *r* = 0.543, *p* = 0.003). Slight positive correlations were also observed between EC and F (Figure 4D, *r* = 0.382, *p* = 0.049) and between VC and phase angle (Figure 4F, *r* = 0.394, *p* = 0.042) of the paretic forearm flexor muscle, moderate negative correlations were observed between NC and X (Figure 4E, *r* = −0.428, *p* = 0.026). No significant correlation was observed between MAS and EIM parameters (*p* > 0.05), and between FMA and any other outcome measures (*p* > 0.05) of the paretic forearm flexor muscle.

### Post Intervention

**Figure 5:**
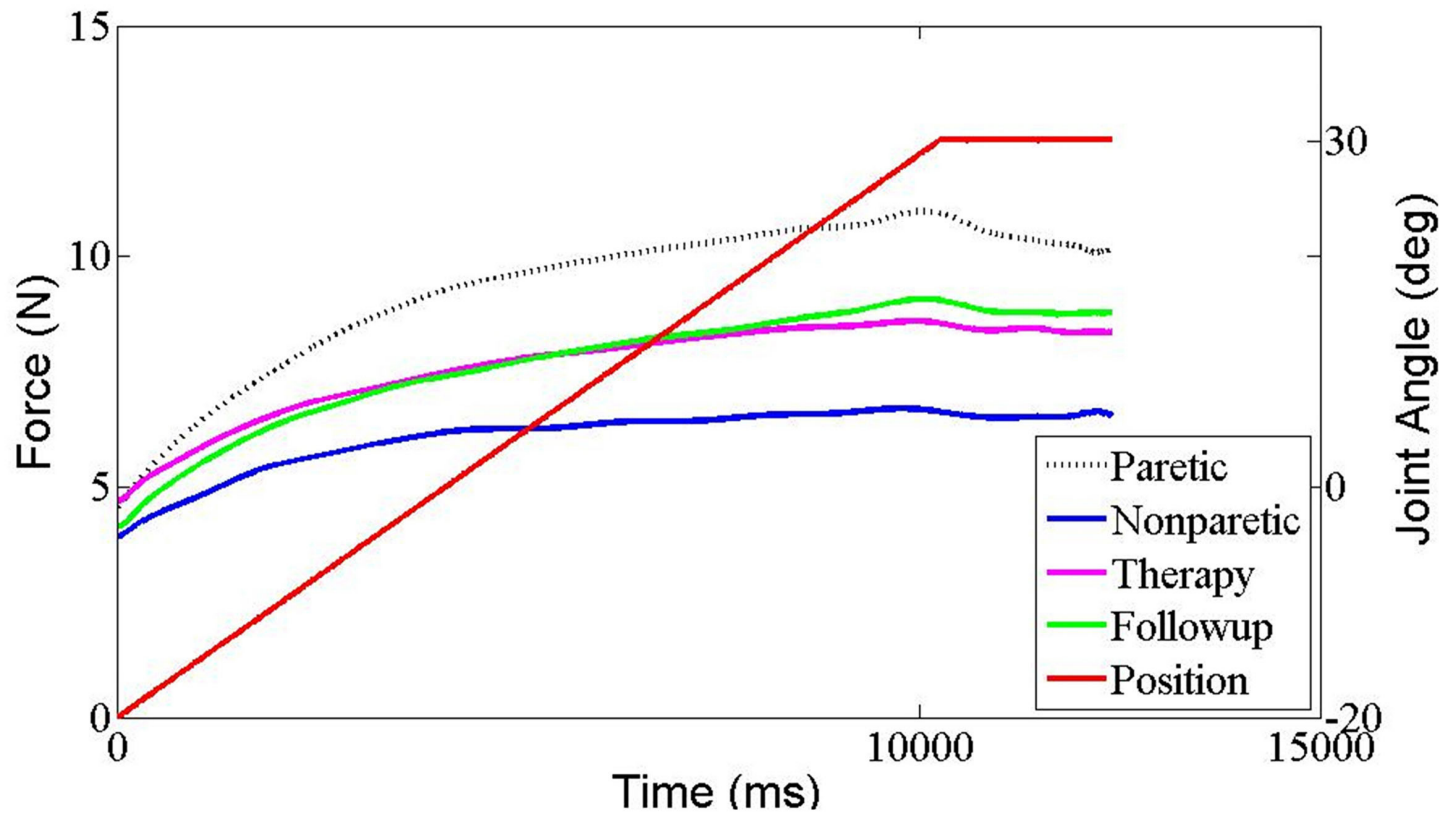
The typical resistant force trend curve in each measurement situation recorded by Neuroflexor in one stroke patient. Red trace represents the angle of wrist movement (from flexion to extension). Black dashed trace represents resistant force of paretic side, Blue trace represents resistant force of non-paretic side, pink trace represents resistant force immediately after ESW intervention of paretic side and green trace represents resistant force at 1 week later of paretic side.

**Figure 6:**
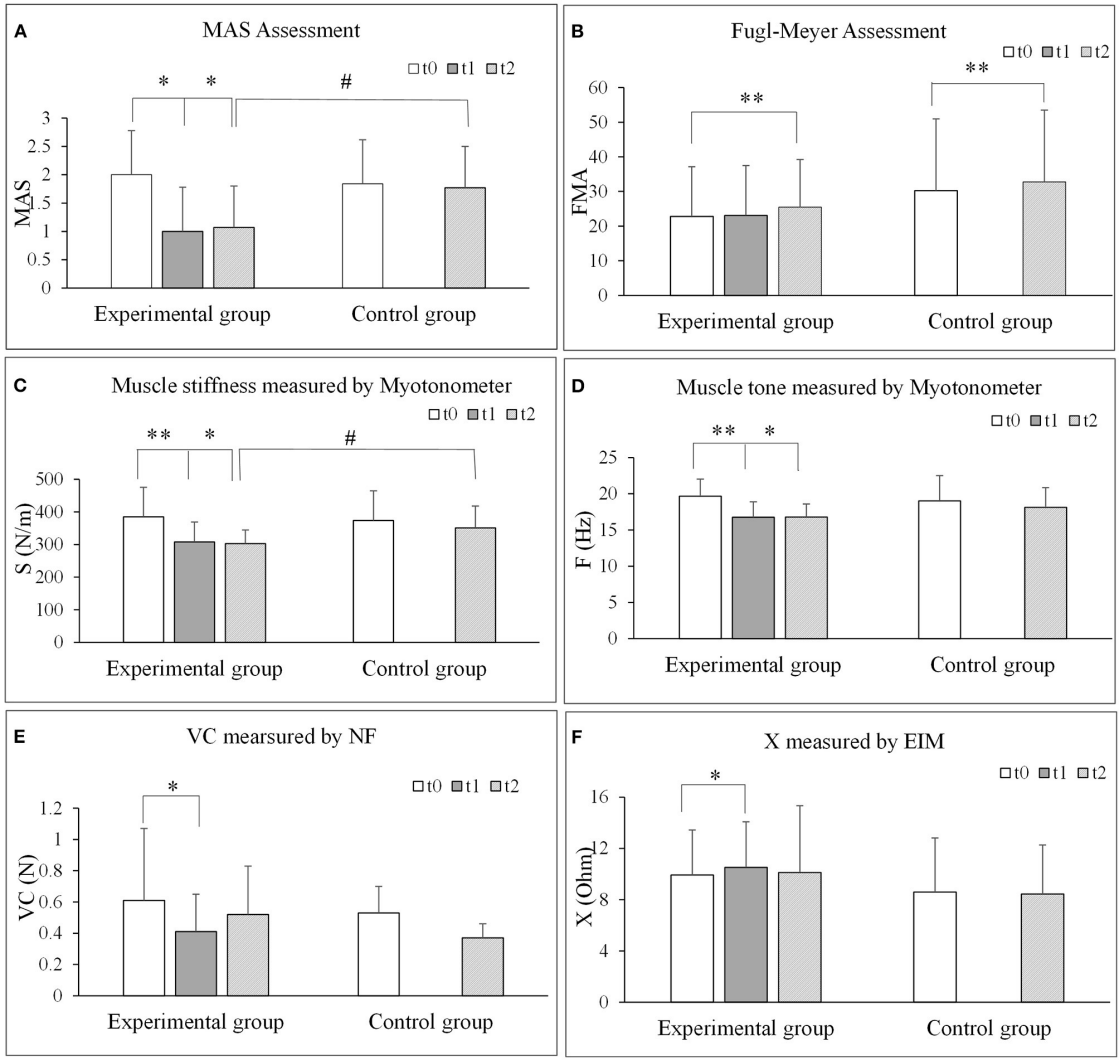
Comparisons of each outcome measures before and after extracorporeal shock wave therapy. **(A)** MAS assessment. **(B)** Fugl-Meyer assessment. **(C)** Muscle stiffness measured by Myotonometer. **(D)** Muscle tone measured by Myotonometer. **(E)** VC measured by NF. **(F)** X measured by EIM. NF, NeuroFlexor; NC, Neural Component; EC, Elasticity Component; VC, Viscosity Component; S, Stiffness; F, Frequency; EIM, Electrical impedance myography; X, Reactance; R, Resistance. *P < 0.05; **P < 0.001.

The results of all the outcome measures at baseline (t0), immediately after rESW (t1) and at the end of the intervention period (t2) are presented in Table 3 and **Figure 6**. The total resistance measured by the NF decreased after intervention compared with pre intervention, but had no significant difference. The typical trend curve is shown in Figure 5. In the intervention group, the MAS score (Figure 6A) significantly decreased immediately after the rESW (t1: *p* = 0.012) and at the end of the intervention period (t2: *p* = 0.021) when compared to baseline. The MAS scores between the experimental group and the control group were significant different at t2 (*p* = 0.030). The FMA scores (Figure 6B) were significantly increased in the experimental group (*p* < 0.001) and the control group (*p* < 0.001) after 1 week of intervention, but the difference between the two group were not significantly different (*p* > 0.05). For the quantitative parameters in the intervention group, S (Figure 6C) and F (Figure 6D) were significantly reduced at t1 (*pS* < 0.001, *pF* < 0.001) and t2 (*pS* = 0.006, *pF* = 0.001) when compared to baseline. VC (Figure 6E) was significantly reduced and X (Figure 6F) was significantly increased at t1 (pVC = 0.033, pX = 0.041) with no significant difference at t2 when compared with t0. NC, EC, R, and phase angle were not significantly different at t1 and t2 compared to baseline. In the control group, all quantitative parameters had no significant difference at t2 when compared with baseline. For the comparison between the experimental group and the control group, S was significantly difference at the end of the intervention period (t2: *p* = 0.034). All other quantitative parameters had no statistically significant difference between the experimental group and control group at the end of intervention.

**Table 3:** All outcome measures at each measuring time point.

**Measurement/Time point**	**t0**	**t1**	**t2**
**MAS**			
Experimental group	2.00± 0.78	1.00± 0.78^*^	1.07± 0.73^*^
Control group	1.84± 0.80		1.77± 0.73
Inter-group *P*-value	0.605		0.030^*^
**Fugl-Meyer**			
Experimental group	22.79 ± 14.37	23.07 ± 14.39	25.50 ± 13.73^**^
Control group	30.23 ± 20.73		32.76 ± 20.73^**^
Inter-group *P*-value	0.285		0.310
**NF**			
VC (N)			
Experimental group	0.61± 0.46	0.41± 0.24^*^	0.52± 0.31
Control group	0.53± 0.17		0.37± 0.09
Inter-group *P*-value	0.533		0.205
EC (N)			
Experimental group	3.63 ± 1.18	3.86 ± 1.60	3.66 ± 2.18
Control group	3.37 ± 0.70		3.92 ± 1.19
Inter-group *P*-value	0.492		0.704
NC (N)			
Experimental group	5.32 ± 5.44	4.75 ± 4.58	4.35 ± 4.49
Control group	3.13 ± 3.65		3.32 ± 3.11
Inter-group *P*-value	0.234		0.499
**Myotonometer**			
F (Hz)			
Experimental group	19.66 ± 2.38	16.76 ± 2.11^**^	16.79 ± 1.81^*^
Control group	19.01 ± 3.49		18.12 ± 2.72
Inter-group *P*-value	0.576		0.147
S (N/m)			
Experimental group	385.50 ± 88.15	308.46 ± 61.09^**^	303.57 ± 42.05^*^
Control group	374.00 ± 97.86		351.46 ± 67.03
Inter-group *P*-value	0.751		0.034^*^
EIM			
X (Ohm)			
Experimental group	9.93 ± 3.51	10.51 ± 3.56^*^	10.11 ± 5.22
Control group	8.59 ± 4.22		8.44 ± 3.83
Inter-group *P*-value	0.378		0.355
R (Ohm)			
Experimental group	50.40 ± 21.49	52.67 ± 22.03	51.76 ± 19.36
Control group	43.84 ± 29.72		44.05 ± 29.38
Inter-group *P*-value	0.515		0.425
Phase angle (Degree)			
Experimental group	9.80 ± 3.23	10.01 ± 3.27	10.03 ± 4.45
Control group	8.71 ± 4.63		8.59 ± 4.62
Inter-group *P*-value	0.485		0.418

*Values of assessments for MAS and Fugl-Meyer scoring, F and S parameters of Myotonometer, VC parameters of NF and X parameters of EIM at the different time points shown as mean ± SD. MAS, Modified Ashworth Score; NF, NeuroFlexor; NC, Neural Component; EC, Elasticity Component; VC, Viscosity Component; S, Stiffness; F, Frequency; EIM, Electrical impedance myography; X, Reactance; R, Resistance*.

* P < 0.05;

** *P < 0.001*.

## Discussion

This study assessed the neural and peripheral contributing factors to post stroke muscle spasticity by applying passive torque measurement combined with biomechanical modeling, myotonometer measurements and electrical impedance myography. This was followed by the investigation of the effect of ESW intervention on muscle spasticity and upper limb function. Significant differences in several factors and mechanical parameters were observed between the affected and unaffected wrist joint of stroke survivors. Biomechanical characteristic parameters (i.e., muscle tone, stiffness, elasticity, and viscosity) and electrical impedance parameters were significantly improved after ESW intervention, especially at immediate effects right after treatment (t1).

### Bilateral Differences and Clinical Relevance

#### NeuroFlexor Method

Some authors stated that although neural reflex hyperexctibility was a key contributing factor to the increase in resistance torque, the reduction in muscle elasticity and increasing in muscle viscosity must also be considered (7, 35). The results of the present study indicated NC, EC, and VC were significantly higher on the paretic side than the non-paretic side. This supports that theory that both the neural factor and the peripheral muscle factor play a role in spasticity. It is possible that different individuals have different portion of contribution from the neural and peripheral components (Leonard et al., 2001; Gäverth et al., 2013; Wang et al., 2017). Previous studies investigated the effect of Botox A injection on spastic elbow flexor using the NeuroFlexor method and reported a significant reduction in the neural factor but not for the non-neural peripheral factor of spasticity (Gäverth et al., 2014; Wang et al., 2018). These results provide evidence to support that individualized intervention is required to address the main spasticity contributing factors in different patients.

#### Muscle Mechanical Properties Measurement

This study observed significant differences in muscle tone and stiffness between the paretic side and non-paretic side as assessed by myotonometer (Table 2). This is consistent with the finding of NeuroFlexor in terms of the involvement of non-neural peripheral muscle factor that contribute to spasticity. The observed differences in muscle tone and stiffness between paretic and non-paretic flexor carpi radialis were consistent with published study which reported differences of 1 Hz and 30 N/m in gastrocnemius in stroke survivors (Park et al., 2019). Our previously published study indicated that spastic the flexor carpi radialis (FCR) tend to have higher stiffness value, as measured by value of shear wave elastography, and the stiffness tend to increase as the stretching angle increased (Leng et al., 2019). These findings add further support the alteration of mechanical properties of spastic muscle post stroke and their contribution to spasticity. Previous studies proposed that changes in biomechanical characteristics after stroke occurrence were related to the changes in muscle morphology, composition and extracellular matrix (Li et al., 2007; Lieber and Ward, 2013). These changes include muscle atrophy, fat infiltration, and increase of fascicular membrane thickness (De Bruin et al., 2014) and a reduction in cross-sectional area and volume (Sions et al., 2012). The movement dysfunction on the affected limb leads to the deposition of extracellular matrix, especially hyaluronic acid (Piehl-Aulin et al., 1991), which in turn contribute to an increase in viscosity between muscle fibers and the difficulty in the sliding of muscle fibers. In the development of the course of disease, if the extracellular matrix deposition is not treated in time, irreversible collagen accumulation will form and lead to increase in muscle fibrosis, muscle stiffness, and reduction in muscle elasticity (Lieber et al., 2003; Raghavan et al., 2016).

#### Electrical Impedance Myography

Previous studies have reported the alteration of muscle impedance of the biceps brachii (Li et al., 2017) and the hypothenar muscle (Zong et al., 2018) after the occurrence of stroke. These findings supported that reactance (X) and phase angle (θ) were stable and sensitive biomarkers in the assessment of muscle intrinsic properties. Resistance and reactance are both related to muscle mass and geometry, as well as tissue quality including extracellular and intracellular water, and the properties of cell membranes (Shiffman et al., 1999). Reduced impedance parameters are related to abnormal muscle fiber structure and damaged membrane integrities, which is induced by the loss of muscle fibers, reduced fiber cross-sectional area, or increased intramuscular extracellular matrix (Rutkove et al., 2002; Li et al., 2014). Metoki et al. (2003) revealed that paretic lower extremity muscles to have an approximate 20% reduction in muscle area and volume compared to the affected side. The contribution of muscle intrinsic property as measured by EIM to spasticity need further investigation.

### Extracorporeal Shock Wave on Spasticity

The results showed that the MAS scores immediately after the ESW intervention (t1) and by the end of the intervention period (t2) were significantly lower, which was consistent with the results of previous studies (Santamato et al., 2013; Troncati et al., 2013; Daliri et al., 2015; Li et al., 2016; Guo et al., 2019). The Fulg-meyer of upper limb scores were significantly increased by the end of the intervention period (t2) in both groups, but no significant difference was observed between groups. This suggested that ESW may not be superior to conventional intervention in promoting the recovery of upper limb motor function. The long-term effect on motor function remains to be studied in the future.

Our results showed that there was a significant decrease in muscle tone, stiffness and viscosity of the wrist flexor on the affected side post ESW intervention (Table 3 and Figure 6). This decreasing corresponds with a significant reduction in MAS score. In previous studies, most reports focused on neuromuscular denervation rather than the structure or biomechanical characteristics of spastic muscle tissue after ESW intervention (Dymarek et al., 2014; Jia et al., 2020). Dymarek et al. (2016b) observed an improvement in trophic condition (electrophysiological and thermal effect) of the spastic muscles post rESW intervention when assessed by infrared thermal (IRT) imaging. Results of the present study observed a reduction in muscle biomechanical characteristics parameters immediately post ESW intervention and continued to be lower by the end of the intervention week. Moon investigated the effects of ESW on spastic muscle by isokinetic dynamometer (Moon et al., 2013). They found that a reduction in the peak eccentric torque, an indicator of muscle stiffness, immediately after rESW intervention and at 1 week follow up. The authors proposed that ESW affects the mechanical muscles stiffness rather than the stretch reflex hyper excitability. Park et al. (2018) investigated the effect of ESW on muscle tone and stiffness with myotonomter in patients with stroke. The study reported a significant reduction of muscle tone (5.1 and 5.7 Hz), and muscle stiffness (17.8 and 22.5 N/m for the muscle flexor carpi radials and flexor digitorum, respectively. The present study observed similar level of reduction of muscle tone and muscle stiffness post ESW. These findings further support that ESW is beneficial in treating spasticity related alteration of muscle mechanical properties.

The VC component of the NeuroFlexor method significantly declined immediately post intervention in the ESW group. Some authors reported that the low energy mechanical vibration induced by the sonic impulse of ESW acts on spastic muscle differently from normal vibrationary stimulation (Romeo et al., 2014). However, the exact mechanism that underpins the alteration of mechanical properties induced by ESW is unclear. The majority of early literature suggested that the benefit of ESW on spasticity was by reducing the hyperexcitability of the alpha motor neuron (Leone and Kukulka, 1988) or the shock wave pressure act on the golgi tendon organ to suppress motor nerve excitability (Bae et al., 2010). However, studies that investigated the underpinning mechanism of ESW by electrophysiological measures of such as Hmax/Mmax (Daliri et al., 2015), F wave (Manganotti et al., 2012), and EMG muscle activities at rest (Dymarek et al., 2014) did not report significant difference in spastic muscle. The neural effects of ESW intervention in spasm are still controversial, recently published systematic review (Dymarek et al., 2014) suggested the neuronal effects were unlikely to be the primarily mechanism to intervene with muscle tone and stiffness since most studies did not observe a reduction in EMG activities (Dymarek et al., 2014). The lack of difference in NC component between the ESW group and the control group observed in the present study adds further support that ESW may address the peripheral muscle factor that contribute to spasticity (Manganotti and Amelio, 2005; Sohn et al., 2011; Santamato et al., 2013). Therefore, it is more likely that the biological response induced by ESW, including increase in blood flow, oxygenation, metabolic process activation and proliferative effect (Notarnicola et al., 2018) may affect the fibrosis and rheological components of muscle tissue, promoting the degradation and absorption of extracellular matrix and decreasing the muscle viscosity and stiffness (Lohse-Busch et al., 2014).

The increase in X after intervention, as measured by EIM, is related to the increase in the number of cell and cell membrane area (Shiffman and Rutkove, 2013). We proposed that the mechanical vibration produced by ESW may result in myofibers to arrange more closely and in more orderly arrangement, which in turn promote the metabolism or redistribution of extracellular matrix, allowing an increase in current resistant passes through under the same unit volume through the myofiber cell member.

### Correlation Between Clinical Scales and Muscle Properties

The present study observed a significant correlation between MAS and NeuroFlexor parameters, and between MAS and myotonometer parameters. MAS score was found to be highly positively correlated with NC value but not the EC and VC values as assessed by the Neuroflexor. This result may reflect the association between the clinical symptoms of spasticity and the stretch reflex excitability (indicated by NC of NeuroFlexor), and the muscle properties (indicated by F and S of myotonometer). The changes observed in the mechanical muscle properties measured by Myotonometer are related to muscle atrophy, fiber composition and extracellular matrix deposition (Aaron et al., 2006). These changes play a role in the increase in resistant torque thus it might reflect the level of spasticity measured by MAS during passive movement. This study provided a novel interpretation about spasticity by combining with muscle intrinsic properties. The correlation between EC (measured by NeuroFlexor) and F (measured by Myotonomter) indicated a positive correlation which provide further support for the feasibility to assess the biomechanical feature of muscle spasticity by these methods.

EIM measured impedance parameters were not correlated to MAS scores (Figure 3). Impedance parameters is reflective of the intrinsic muscle properties which include the quality of cell membranes and connective tissues of the muscles. The lack of association may be related to the core of MAS measurement that is to assess the joint resistant during passive movement (Bohannon and Smith, 1987), while the EIM parameters measured the intrinsic muscle properties of a particular muscle group. In addition, the MAS is suggested to be more related to an increase in neural stretch reflex activity (Malhotra et al., 2009; Fleuren et al., 2010) rather than considering the non-neural mechanical properties of the resistance (Dietz and Sinkjaer, 2007). There is no significant correlation between FMA and muscle intrinsic properties as measured by the NeuroFlexor, myotonometer or EIM. FMA is more related to motor functional recovery of the entire upper limb, rather than the function of a single muscle group. Thus, in future study, a technique that focused on function assessment after intervention of one particular muscle such as sEMG, shear wave elasticity should be correlated to motor function to further reveal the correlation between muscle function and intrinsic properties.

## Limitations

The findings of the present study should be interpreted with cautious due to its limitations. First, this study administered ESW for a single session (Sohn et al., 2011; Santamato et al., 2014) and outcome measures were recorded by the end of the 1-week intervention period. Thus, it is unclear the optimal intensity and frequency of ESW intervention, or if the observed benefit may persist through medium long term. In addition, the lack of a placebo treatment to compare with rESW is another limitation of this study to interpretate post intervention changes. Since the placebo effects may not be null, we will include the sham rESW as previous studies (Manganotti and Amelio, 2005; Daliri et al., 2015) in the future study. The contribution of neural factor was assessed by the NC parameters of the NeuroFlexor, which was based on published biomechanical modeling method (Lindberg et al., 2011; Wang et al., 2017). Further studies that involve other means to assess neural activities, such as EMG on F-wave and H-flex, is recommended to substantiate the findings of ESW on muscle spasticity. This study could not ascertain if the tested muscle was completely relaxed during myotonometer assessment, despite the wrist joint was placed in a neutral or apparently relaxed position. While devices such as EMG could assess the muscle relaxation state, it was not be feasible to simultaneously apply EMG and myotonometer. This was due to the placement of the electrodes on the skin surface would affect the myotonometer's measurements. Last, the lack of pain assessment is another limitation of this study since pain was reported to have a reciprocal connection with spasticity that may also be affected by rESW.

## Conclusion

This study reported quantifiable changes in upper limb muscle properties in post stroke muscle spasticity which adds further support to the theory that both neural component and peripheral component play a role in muscle spasticity. ESW intervention may be more effective in addressing the peripheral component of spasticity. The clinical management of post stroke spasticity should consider both the neural and non-neural factors in order to identify optimal intervention regime.

## Data Availability Statement

The datasets analyzed during the current study are available from the corresponding authors upon reasonable request.

## Ethics Statement

The studies involving human participants were reviewed and approved by the Ethics Committee of the First Affiliated Hospital, Sun Yat-sen University [Ethics Number: (2017).143]. The patients/participants provided their written informed consent to participate in this study.

## Author Contributions

YL, LL, and DH conceived and designed the study. YL, CH, RB, and ZX performed the experiments. YL and WL wrote the paper. WL, XS, DH, and LL made contributions to the experiments. WL, DH, and LL reviewed and edited the manuscript. All authors had read and approved the manuscript.

## Conflict of Interest

The authors declare that the research was conducted in the absence of any commercial or financial relationships that could be construed as a potential conflict of interest.
